# Hyperactivation of mTORC1 disrupts cellular homeostasis in cerebellar Purkinje cells

**DOI:** 10.1038/s41598-019-38730-4

**Published:** 2019-02-26

**Authors:** Yusuke Sakai, Hidetoshi Kassai, Hisako Nakayama, Masahiro Fukaya, Tatsuya Maeda, Kazuki Nakao, Kouichi Hashimoto, Hiroyuki Sakagami, Masanobu Kano, Atsu Aiba

**Affiliations:** 1 Laboratory of Animal Resources, Center for Disease Biology and Integrative Medicine, Graduate School of Medicine, The University of Tokyo, Tokyo, 113-0033 Japan; 2 Department of Neurophysiology, Graduate School of Medicine, The University of Tokyo, Tokyo, 113-0033 Japan; 3 Department of Neurophysiology, Graduate School of Biomedical and Health Sciences, Hiroshima University, Hiroshima, 734-8551 Japan; 4 Department of Anatomy, Kitasato University School of Medicine, Sagamihara, 252-0374 Japan; 5 Institute of Molecular and Cellular Biosciences, The University of Tokyo, Tokyo, 113-0032 Japan; 6 International Research Center for Neurointelligence (WPI-IRCN), The University of Tokyo Institutes for Advanced Study (UTIAS), The University of Tokyo, Tokyo, 113-0033 Japan; 7 Present Address: Department of Physiology I (Neurophysiology), School of Medicine, Tokyo Women’s Medical University, Tokyo, 162-8666 Japan; 8 Present Address: Department of Biology, Hamamatsu University School of Medicine, Hamamatsu, Shizuoka, 431-3192 Japan

## Abstract

Mammalian target of rapamycin (mTOR) is a central regulator of cellular metabolism. The importance of mTORC1 signaling in neuronal development and functions has been highlighted by its strong relationship with many neurological and neuropsychiatric diseases. Previous studies demonstrated that hyperactivation of mTORC1 in forebrain recapitulates tuberous sclerosis and neurodegeneration. In the mouse cerebellum, Purkinje cell-specific knockout of *Tsc1/2* has been implicated in autistic-like behaviors. However, since TSC1/2 activity does not always correlate with clinical manifestations as evident in some cases of tuberous sclerosis, the intriguing possibility is raised that phenotypes observed in *Tsc1/2* knockout mice cannot be attributable solely to mTORC1 hyperactivation. Here we generated transgenic mice in which mTORC1 signaling is directly hyperactivated in Purkinje cells. The transgenic mice exhibited impaired synapse elimination of climbing fibers and motor discoordination without affecting social behaviors. Furthermore, mTORC1 hyperactivation induced prominent apoptosis of Purkinje cells, accompanied with dysregulated cellular homeostasis including cell enlargement, increased mitochondrial respiratory activity, and activation of pseudohypoxic response. These findings suggest the different contributions between hyperactivated mTORC1 and *Tsc1/2* knockout in social behaviors, and reveal the perturbations of cellular homeostasis by hyperactivated mTORC1 as possible underlying mechanisms of neuronal dysfunctions and death in tuberous sclerosis and neurodegenerative diseases.

## Introduction

Mammalian (or mechanistic) target of rapamycin (mTOR) is an evolutionarily conserved protein kinase that acts as two functionally distinct complexes, termed mTORC1 and mTORC2^1^. mTORC1 signaling serves as a central hub for the regulation of cellular metabolism, integrating various environmental stimuli such as growth hormones and amino acids^2^. Activation of mTORC1 enhances protein synthesis, while inhibiting autophagy, and dysregulated activation of mTOR is implicated in many human diseases like cancer and diabetes. In the central nervous system, mTOR signaling is involved in neuronal development including cell migration and synaptic plasticity^3^. Since the brain is one of the most energy-consuming organs, the importance of mTORC1 signaling is emphasized from the standpoint of understanding neurological and neuropsychiatric disorders^4^. Animal models of mTOR-related diseases have been established by activating mTORC1 signaling in specific regions of the brain. Forebrain-specific activation of mTORC1 signaling clearly recapitulates tuberous sclerosis and neurodegeneration^5,6^. However, relationship between these neurological manifestations and mTOR signaling in other brain regions remains unclear.

The cerebellum controls motor coordination and motor learning^7–9^. The Purkinje cell is the only output neuron in the cerebellar cortex that receives two distinct excitatory inputs from parallel fibers (PFs) and climbing fibers (CFs). In the neonatal cerebellum, the Purkinje cell is innervated by multiple CFs and surplus CFs are gradually eliminated to establish mono-innervation in adulthood^10^. Both motor coordination and synapse elimination are hallmarks of Purkinje cell functions, and many synaptic proteins are involved in these processes^10^. Recent studies demonstrate that the cerebellum is also implicated in higher cognitive functions^11^, and atrophied cerebellum and loss of Purkinje cells have been found in some patients with autism spectrum disorder (ASD)^12^. Considering that modulators of mTOR signaling such as PTEN and FMR1 are responsible genes of ASD, dysregulated mTOR signaling in Purkinje cells may be linked to this disorder.

Animal models of mTOR-related diseases in the cerebellum have been established by deleting *Tsc1* or *Tsc2* gene specifically in Purkinje cells. TSC1 and TSC2 form a complex and negatively regulate mTORC1 activity acting as GTPase activating protein (GAP) of Rheb. Purkinje cell-specific *Tsc1/2* knockout mice exhibit abnormal behaviors in social interaction test, suggesting that aberrant activation of mTORC1 in Purkinje cells may be responsible for the onset of ASD-like symptoms. However, mTORC1 activity is modulated by many regulatory molecules, the phenotypes observed in *Tsc1/2* knockout mice should not be attributed solely to mTORC1 hyperactivation. In fact, human patients with N525S in TSC2 display severe symptoms of tuberous sclerosis without affecting TSC1/2 complex formation or GAP activity toward Rheb, whereas G1556S mutation impairs GAP activity with mild symptoms^13,14^. These clinical cases raise the possibility that activity of mTORC1 signaling does not correlate with symptom severity in some cases of tuberous sclerosis.

In the present study, to address mTORC1-specific contribution in cerebellar functions, we generated transgenic (Tg) mice in which mTORC1 signaling is directly activated in Purkinje cells by using hyperactive mTOR mutant. Surprisingly, we did not find any abnormality in social behavior in our Tg mice, suggesting that activation of mTORC1 in Purkinje cells is insufficient for the onset of ASD-like symptoms. On the other hand, these Tg mice exhibited motor discoordination accompanied with pronounced apoptosis and impaired synapse elimination of Purkinje cells. Furthermore, hyperactivated mTORC1 signaling induced increased cell size, pseudohypoxic state and abnormal mitochondrial dynamics. Our findings provide evidence that mTORC1 signaling in Purkinje cells is important for maintenance of cellular homeostasis.

## Results

### Activation of mTORC1 in cerebellar Purkinje cells

To investigate physiological roles of mTORC1 signaling in cerebellar Purkinje cells, we used hyperactive mTOR in which four point mutations are introduced in the rat mTOR gene^15^. Hyperactive mTOR can retain its kinase activity toward the mTORC1 pathway even under the starvation condition in the cultured cells^15^ and brains^5^. For activation of the mTORC1 pathway in Purkinje cells, hyperactive mTOR gene was placed under the control of TRE promoter (TRE-mTOR Tg)^5^, and expression of tTA was driven by L7 promoter, which leads to expression of active mTOR in Purkinje cells (L7-tTA Tg, Supplementary Fig. S1a)^16^. Therefore, hyperactive mTOR expression can be controlled by doxycycline administration. In this study, we established homozygous double Tg mice (*TRE-mTOR*^*SL1+IT*^*/TRE-mTOR*^*SL1+IT*^*; L7-tTA/L7-tTA*) for adequate expression of hyperactive mTOR (refer as PC-mTOR Tg mice hereafter). Homozygous double Tg mice in which expression of hyperactive mTOR was suppressed by doxycycline administration were used as experimental control animals.

**Figure 1:**
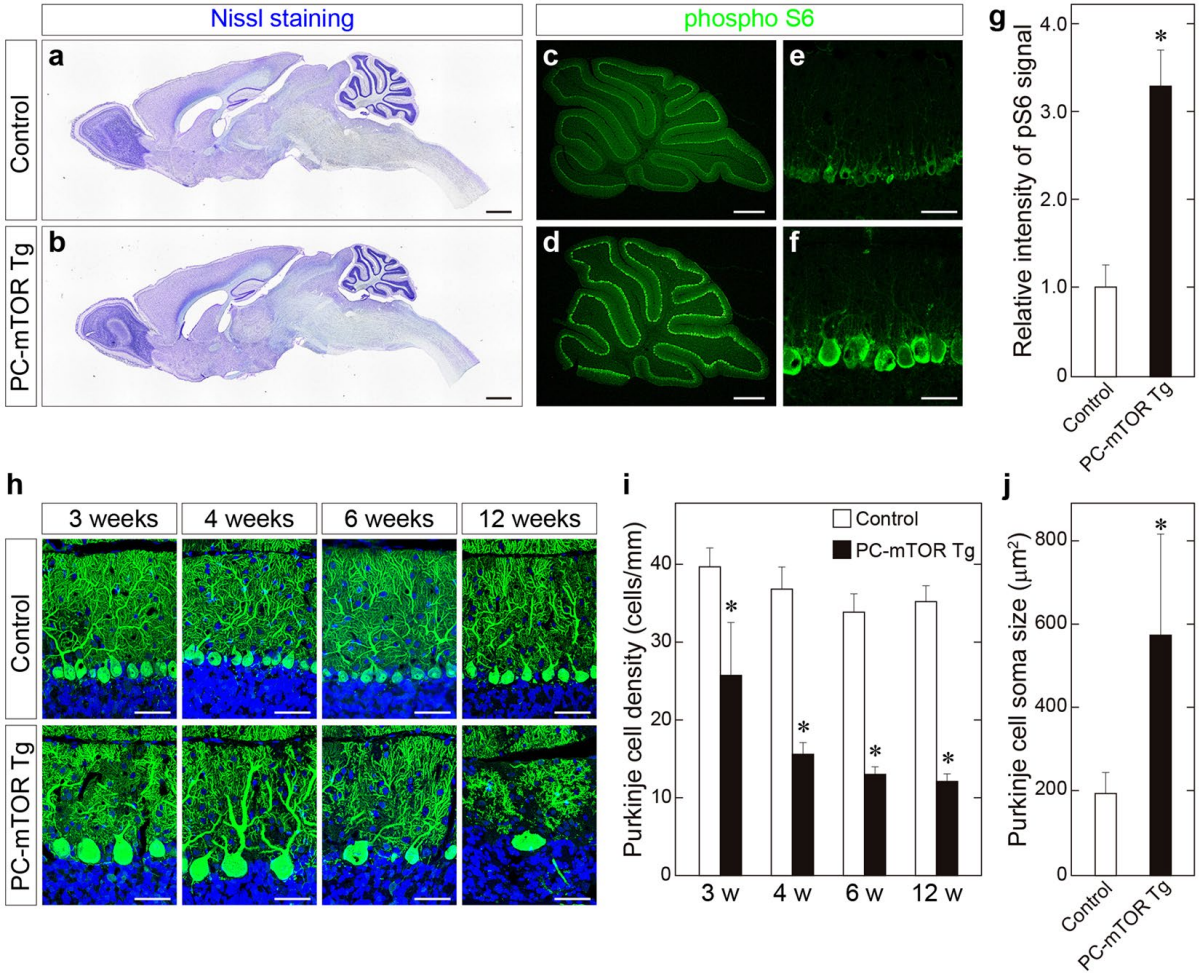
Activation of mTORC1 signaling in Purkinje cells of PC-mTOR Tg mice. (**a**,**b)** Brain morphology of control and PC-mTOR Tg mice at 12 weeks of age. Parasagittal brain sections were stained with cresyl violet. Overall brain morphology was preserved in PC-mTOR Tg mice except the atrophied cerebellum. (**c**–**g**) Enhanced phosphorylation of S6 protein in Purkinje cells of PC-mTOR Tg mice. The sagittal sections from control (**c** and **e**) and PC-mTOR Tg (**d** and **f**) cerebella at 4 weeks of age were immunostained with an antibody to phosphorylated S6 protein. Immunopositive signals were quantified in panel g. (**h–j**) Age-dependent changes of cerebellar morphology of control and PC-mTOR Tg mice. In PC-mTOR Tg mice, Purkinje cell density was significantly decreased with age, and hypertrophied Purkinje cells were obvious from 3 weeks of age. The density and soma area of Purkinje cells were quantified in panel i and j, respectively. Scale bars, 1 mm (**a** and **b**); 500 μm (**c** and **d**); 50 μm (**e**,**f** and **h**). *p < 0.001 by Student *t*-test (**g**,**i** and **j**); control, n = 12 cells from 2 mice; PC-mTOR Tg, n = 12 cells from 2 mice (**g**); n = 10 slices from 2 mice at each time point (**i**); control, n = 101 cells from 2 mice; PC-mTOR Tg, n = 133 cells from 2 mice (**j**).

PC-mTOR Tg mice are viable and fertile with no overt abnormality in their appearances. We first observed the brain morphology of PC-mTOR Tg mice. Nissl staining of the brain slices showed that the cerebellum of PC-mTOR Tg mice was smaller than that of the control in adulthood, while other brain regions were indistinguishable between them (Fig. 1a and b). The molecular layer of the cerebellar cortex of PC-mTOR Tg mice was thinner than that of the control (Fig. 1h, Supplementary Fig. S4c), probably due to the atrophy of the cerebellum. Next, we examined expression of hyperactive mTOR in the PC-mTOR Tg cerebella. Immunoblot analysis revealed that hyperactive mTOR protein could be detected at 2 weeks of age in PC-mTOR Tg mice (Supplementary Fig. S1b). Subsequently, hyperactive mTOR expression was gradually decreased, and was almost undetectable at 6 weeks of age (Supplementary Fig. S1b). We confirmed activation of the mTORC1 pathway in Purkinje cells by immunohistochemical analysis. Since ribosomal S6 protein is a substrate of p70 S6 kinase, it is often used as an *in vivo* readout of mTORC1 activity. Phosphorylation of S6 protein was enhanced in cell bodies of Purkinje cells in PC-mTOR Tg mice compared to the control (Fig. 1c–g), indicative of Purkinje cell-specific hyperactivation of mTORC1 signaling in PC-mTOR Tg mice.

**Figure 2:**
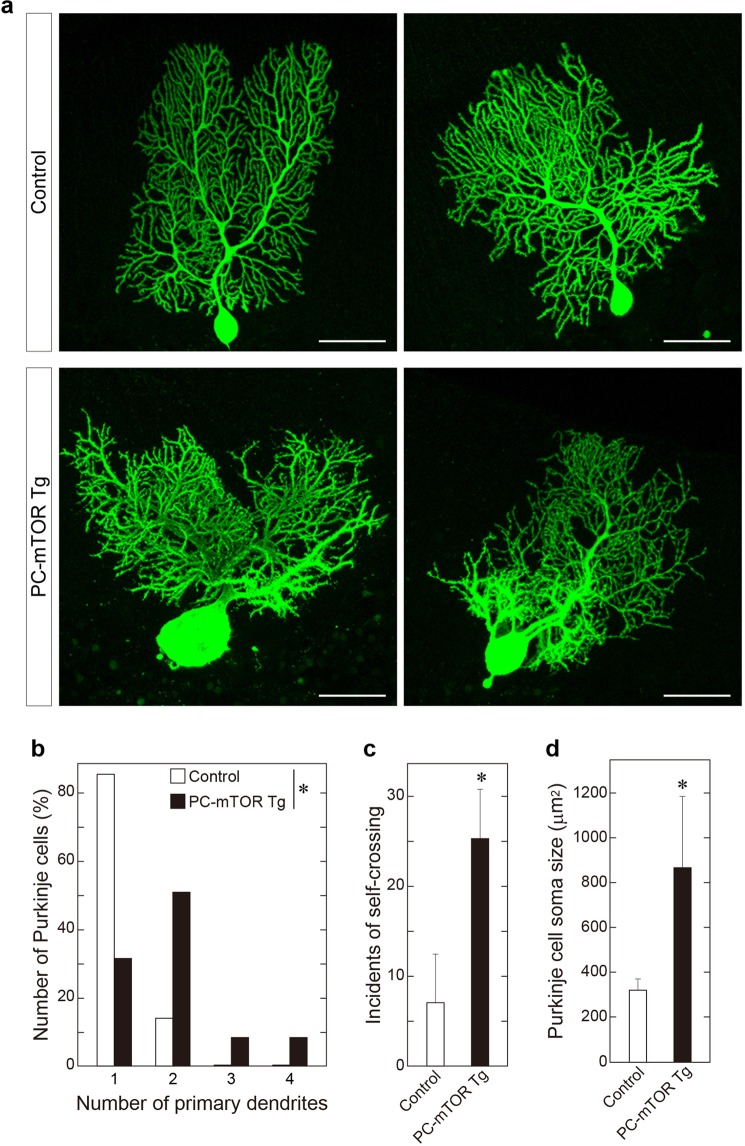
Morphological analysis of single Purkinje cells. (**a**) Purkinje cell morphology at single cell resolution. Biocytin was microinjected into a single Purkinje cell of a cerebellar slice, and stained with streptavidin conjugated with Alexa 488. Scale bars, 50 μm. (**b**) Increased number of primary dendrites in PC-mTOR Tg mice. In control mice, the majority of Purkinje cells had a single primary dendrite that was originated from cell bodies. In contrast, Purkinje cells with more than two primary dendrites were frequently found in PC-mTOR Tg mice. *p < 0.001 by Mann-Whitney Rank Sum test; control, n = 70 cells; PC-mTOR Tg, n = 47 cells. (**c**) Impaired dendritic tiling in PC-mTOR Tg mice. The number of self-crossing of distal dendrites was counted in the control and PC-mTOR Tg cells (per 3946.4 μm^2^ square area). Self-crossing was significantly increased in PC-mTOR Tg mice, indicating that mTORC1 signaling may be important for the dendritic arborization. (**d**) Enlarged Purkinje cell bodies in PC-mTOR Tg mice. The soma areas were significantly expanded in PC-mTOR Tg mice, consistent with the data shown in Fig. 1j. Values are means ± SD. *p < 0.001 by Student *t*-test (**c** and **d**); control, n = 90 areas from 6 mice; PC-mTOR Tg, n = 15 areas from 6 mice (**c**); control, n = 38 cells from 6 mice; PC-mTOR Tg, n = 37 cells from 6 mice (**d**).

We examined time-dependent changes in cerebellar morphology in PC-mTOR Tg mice. Purkinje cells in cerebellar slices were stained with anti-calbindin antibody at ages ranging from 3 to 12 weeks (Fig. 1h). In PC-mTOR Tg mice, cell bodies and dendrites of Purkinje cells were immensely enlarged from 3 weeks of age (Figs 1h,j and 2d). Number of Purkinje cells was already decreased at 3 weeks of age in PC-mTOR Tg mice, and further declined with age at about 30% of that of the control (Fig. 1h and i). For more detailed morphological analysis, we observed Purkinje cells at single-cell resolution by injecting biocytin tracer, and quantitatively analyzed the dendrite morphology (Fig. 2a). For the number of the primary dendrites, more than 80% of control Purkinje cells had a single dendrite, whereas the majority of PC-mTOR Tg cells had more than two (Fig. 2b). Distal dendrites of Purkinje cells usually develop non-overlapping patterns by self-avoidance, which is required to maximize their receptive fields^17^. Hyperactivation of mTORC1 in Purkinje cells caused an excessive arborization of the distal dendrites and a strong increase in self-crossings of the branched dendrites (Fig. 2c). Purkinje cell-specific Rictor knockout mice also show increased number of primary dendrites and self-crossings^18^, implying that feedback inhibition of mTORC2 pathway by mTORC1 hyperactivation may underlie these phenotypes (see Discussion).

**Figure 3:**
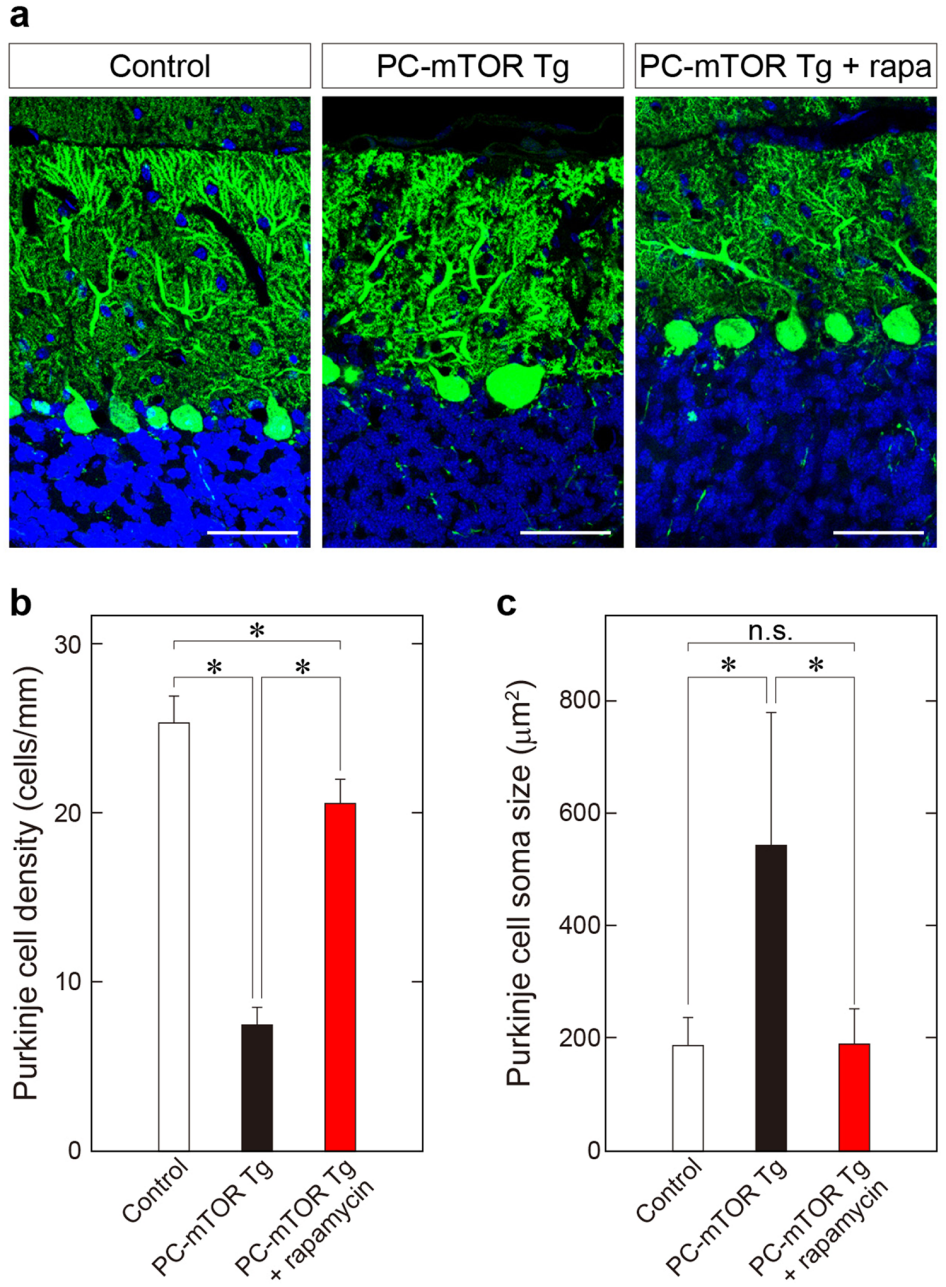
Rescue of decreased cell number and hypertrophy of Purkinje cells in PC-mTOR Tg mice by administration of rapamycin. (**a**) Morphology of Purkinje cells stained with calbindin antibody. Intraperitoneal administration of rapamycin to PC-mTOR Tg mice at 3–6 weeks of age dramatically suppressed both hypertrophy of cell body and reduction of Purkinje cell density. Scale bars, 50 μm. (**b**,**c**) Quantification of density and soma size of Purkinje cells from the control, PC-mTOR Tg and Tg mice that received rapamycin injection. These results indicate that Purkinje cell phenotypes observed in PC-mTOR Tg mice are attributable to activation of mTORC1 signaling. *p < 0.001 by one-way ANOVA with Tukey; control, n = 15 slices from 3 mice; PC-mTOR Tg, n = 9 slices from 2 mice; PC-mTOR Tg + rapamycin, n = 10 slices from 2 mice (**b**); control, n = 188 cells from 3 mice; PC-mTOR Tg, n = 71 cells from 2 mice; PC-mTOR Tg + rapamycin, n = 194 cells from 2 mice (**c**). n.s., not significant.

We tested whether the hypertrophy and the decreased number of Purkinje cells observed in PC-mTOR Tg mice can be attributable solely to mTORC1 hyperactivation by the rescue experiment using rapamycin. Rapamycin is a specific inhibitor toward mTORC1 signaling, inhibiting phosphorylation of p70 S6 kinase. As expected, phosphorylation of S6 protein in Purkinje cells was significantly decreased in PC-mTOR Tg mice after administration of rapamycin (Supplementary Fig. S2a). Both hypertrophy and decreased number of Purkinje cells were strikingly reversed in PC-mTOR Tg mice by the rapamycin treatment, almost comparable to the control mice (Fig. 3). These data demonstrates that mTORC1 signaling in Purkinje cells is essential for cell size regulation and cell survival, and may play an important role in neuronal self-recognition during the dendritic arborization.

### Impaired motor coordination in PC-mTOR Tg mice

Since *Tsc1/2* knockout mice show abnormal behavioral phenotypes such as motor discoordination and autistic-like social deficits^19,20^, we performed a series of behavioral tests using PC-mTOR Tg mice. The open field test is widely applied to quantify locomotor activity and anxiety-like behavior in rodents. In PC-mTOR Tg mice, both total time and distance spent in exploratory activities were significantly reduced compared to the control (Supplementary Fig. S3a). In contrast, no differences were detected in time spent in the center or periphery of the open field between control and PC-mTOR Tg mice. These data suggest that mTORC1 activation in Purkinje cells affects the locomotor activity but not anxiety-like behavior. Next, we examined the impact of hyperactivation of mTORC1 signaling on social behaviors using the three chamber test (Supplementary Fig. S3b). During the habituation period, both control and PC-mTOR Tg mice showed no preference for the right and left chamber. When a stranger mouse was put in the left chamber, control mice stayed for a significantly longer time in the left chamber with a stranger mouse than in the right side chamber with an empty cage. These trends were also observed in PC-mTOR Tg mice, displaying the normal level of social novelty recognition even under the condition of mTORC1 hyperactivation in Purkinje cells.

**Figure 4:**
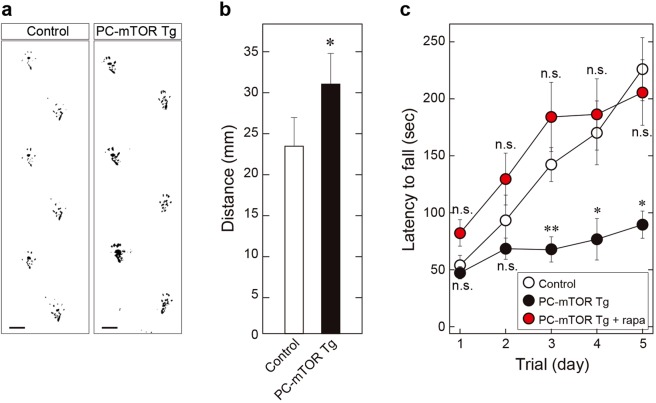
Gait analysis and rotarod test of PC-mTOR Tg mice. (**a**,**b**) Footprint patterns of control and PC-mTOR Tg mice. In PC-mTOR Tg mice, distance between left and right hind paw traces was significantly wider than the control, indicative of the ataxic gait. Scale bars, 1 cm. *p < 0.001 by Student *t*-test; control, n = 6; PC-mTOR Tg, n = 5. (**c**) Rotarod test of 6-week-old mice. An accelerating rotarod was used to measure the motor coordination and motor learning of mice. Retention time on the rotating rod was significantly lower in PC-mTOR Tg mice (close circles) then the control (open circles) over the course of multiple training days. Administration of rapamycin to PC-mTOR Tg mice drastically rescued the motor discoordination (red circles) without any significant differences from control mice. Values are mean ± SEM of three consecutive trials. *p < 0.001 and **p < 0.05 by two-way ANOVA with Tukey for both PC-mTOR Tg and PC-mTOR Tg + rapamycin compared to control; control, n = 5; PC-mTOR Tg, n = 6; PC-mTOR Tg + rapamycin, n = 5. n.s., not significant.

Given that PC-mTOR Tg showed the atrophied cerebellum that accompanied Purkinje cell death, we examined motor function by performing the footprint analysis and the rotarod test. At 12 weeks of age, PC-mTOR Tg mice apparently exhibited a moderate ataxic gait, as shown by the footprint patterns of the hindlimb (Fig. 4a). PC-mTOR Tg mice showed irregular step patterns with a significantly broader gait width compared with control mice (Fig. 4b). Quantitative analysis of motor coordination and motor learning was performed using the rotarod test. PC-mTOR Tg mice showed a significant deficit in the ability to maintain balance on the rotating rod compared to the control (Fig. 4c). Hypoactivity of PC-mTOR Tg mice in the open field test may be partially attributable to the motor discoordination. Although retention time on the rod was increased day by day in control mice, only slight improvement was observed in PC-mTOR Tg mice (Fig. 4c). Furthermore, three-week administration of rapamycin before the rotarod test strikingly rescued both motor discoordination and impaired motor learning in PC-mTOR Tg mice (Fig. 4c). Together, these results indicate that hyperactivation of mTORC1 signaling in Purkinje cells impairs not only motor coordination but also motor learning without affecting social behaviors.

### Reduced excitability and multiple CF-innervation of Purkinje cells in PC-mTOR Tg mice

**Figure 5:**
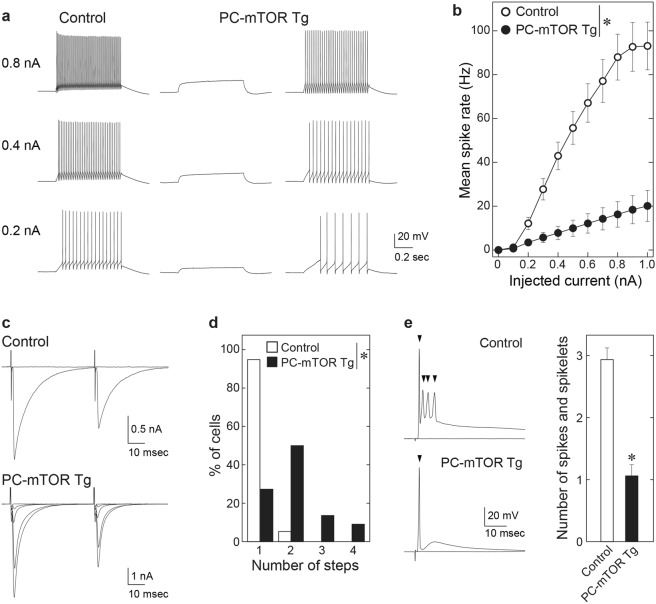
Electrophysiological properties of Purkinje cells from control and PC-mTOR Tg mice. (**a**) Specimen traces of voltage responses to depolarizing current injection of 0.2 nA (bottom), 0.4 nA (middle), and 0.8 nA (top) recorded in a control Purkinje cell (left), a PC-mTOR Tg Purkinje cell without firing (middle) and a PC-mTOR Tg Purkinje cell generating action potentials (right). Depolarizing currents were applied from −65 mV. (**b**) Summary of mean spike rates in response to current commands from 0 to 1.0 nA. The firing rate was significantly lower in PC-mTOR Tg than control mice. *p < 0.001 by two-way repeated measures ANOVA; control, n = 30; PC-mTOR Tg, n = 24. (**c**) Specimen traces of CF-EPSCs in response to paired stimuli with 50 ms interval in a control (upper) and a PC-mTOR Tg Purkinje cell (lower). Holding potential (Vh) was −10 mV. (**d**) Summary histogram showing the number of discrete CF-EPSC steps in Purkinje cells from control (n = 38 cells from 4 mice) and PC-mTOR Tg mice (n = 22 cells from 3 mice) showed significant difference (*p < 0.001 by Mann-Whitney Rank Sum test). (**e**) CF-EPSP in a control (upper) and a PC-mTOR Tg Purkinje cell (bottom) elicited by stimulating a single CF that elicited the largest EPSCs in each Purkinje cell. Vh = −65 mV. The histograms represent the numbers of spikes and spikelets (control, n = 17; PC-mTOR Tg, n = 14). The number was significantly smaller in PC-mTOR Tg than control (*p < 0.001 by Mann-Whitney Rank Sum test; control, 2.93 ± 0.20, n = 14; PC-mTOR Tg, 1.06 ± 0.18, n = 17).

Motor dysfunction of PC-mTOR Tg mice prompted us to investigate electrophysiological properties of Purkinje cells. In control mice, 67% of Purkinje cells (n = 20/30) showed spontaneous firing at the resting membrane potential in the slice preparation. By marked contrast, only 9% of Purkinje cells in PC-mTOR Tg mice (n = 2/22) fired spontaneously (Table 1, Spontaneous spike rate), although resting membrane potentials were normal level (Table 1, V_rest_). This trend was also observed in the spike generation elicited by current injection. All Purkinje cells in control mice generated action potentials in response to depolarizing current pulses. On average, mean spike rate increased with injected current amplitudes. In contrast, spike generation was severely impaired in PC-mTOR Tg mice (Fig. 5a and b). Many Purkinje cells (n = 7/24) failed to generate spikes by maximum current injections that we tested (1000 pA) in PC-mTOR Tg mice. Such low excitability may be partially attributable to the low input resistance in PC-mTOR Tg mice (Table 1, Input resistance). These data suggest that membrane excitability was greatly lowered in PC-mTOR Tg mice.

**Table 1:** Membrane properties of Purkinje cells.

	Control	n	PC-mTOR Tg	n	*p* value
V_rest_ (mV)	−52.8 ± 0.9	30	−54.4 ± 1.6	19	0.359
Input resistance (MΩ)	46.0 ± 1.4	29	30.3 ± 1.9	24	<0.001
Spontaneous spike rate (Hz)	22.7 ± 4.1	30	2.4 ± 1.7	22	<0.001

Statistical analyses were performed with the Student *t*-test for V_rest_ and Input resistance, and with Mann-Whitney Rank Sum test for Spontaneous spike rate.

Next, we examined whether synaptic transmission was affected by hyperactivated mTOR signaling in Purkinje cells. In most Purkinje cells of control mice, only one CF-EPSC with large amplitude was elicited in an all-or-none manner as stimulus intensity was gradually increased (n = 36/38, left upper traces in Fig. 5c). In contrast, the amplitude of CF-EPSC was increased in a stepwise manner in 73% of PC-mTOR Tg Purkinje cells (n = 16/22, left bottom traces in Fig. 5c). These data suggest that almost all Purkinje cells are innervated by single CFs in control mice^21,22^, whereas the majority of them receive inputs from multiple CFs in PC-mTOR Tg mice (Fig. 5d). In addition to the multiple CF-innervation, the kinetics of CF-EPSCs was also affected in PC-mTOR Tg mice. CF-EPSCs in Tg mice showed significantly larger amplitude (Table 2, Amplitude), slower 10–90% rise time (Table 2, Rise time) and faster decay time constant (Table 2, τ_decay_), while the paired-pulse ratio was normal (Table 2, Paired-pulse ratio). For histological confirmation of abnormal CF innervation in PC-mTOR Tg mice, the cerebellar slices were immunostained with VGluT2 antibody. VGluT2 is known to be expressed in CF terminals that are identified as large puncta on Purkinje cell dendrites^23^. CF terminals penetrated over 80% of the molecular layer thickness in control mice, whereas the penetration of CF terminals was significantly less in PC-mTOR Tg mice (Supplementary Fig. S4d). Together, these data support the notion that mTOR signaling in Purkinje cells plays an important role in establishment of mono-innervation and proper synaptic transmission by CFs to Purkinje cells.

**Table 2:** Kinetics of CF- and PF-EPSCs.

		Control	n	PC-mTOR Tg	n	*p* value
CFs mono & multi-S	Amplitude (pA)	1605 ± 70	32	2745 ± 185	21	<0.001
	Rise time (ms)	0.51 ± 0.02	32	0.62 ± 0.03	19	<0.001
	τ_decay_ (ms)	8.89 ± 0.22	32	4.85 ± 0.31	19	<0.001
	Paired-pulse ratio	0.71 ± 0.01	32	0.67 ± 0.02	21	0.051
PFs	Paired-pulse ratio	1.74 ± 0.03	39	1.65 ± 0.06	14	0.115

Statistical analyses were performed as follows; Mann-Whitney Rank Sum test for Amplitude, Rise time and Paired-pulse ratio; Student *t*-test for τ_decay_. Kinetics and paired-pulse ratio of CF-EPSCs were measured from CFs sampled from mono-innervated Purkinje cells (mono) and CFs with the largest EPSC amplitude in multiply-innervated Purkinje cells (multi-S).

We examined how output voltage responses of Purkinje cells to CF inputs were affected by the reduced membrane excitability and altered CF-EPSCs in PC-mTOR Tg mice. The EPSPs evoked by the strongest CF in each Purkinje cell were recorded at −65 mV. In control Purkinje cells, CF stimulation elicited complex spikes composed of a single overshooting full spike followed by a few spikelets (left upper traces in Fig. 5e). However, only an action potential without spikelets was elicited in most Purkinje cells of PC-mTOR Tg mice (left bottom in Fig. 5e). The numbers of spikes and spikelets was significantly smaller in PC-mTOR Tg than control mice (Fig. 5e). These results suggest that information transfer to the deep cerebellar nuclei via CFs is severely disturbed in PC-mTOR Tg mice.

### Induction of cellular stresses by mTORC1 hyperactivation

**Figure 6:**
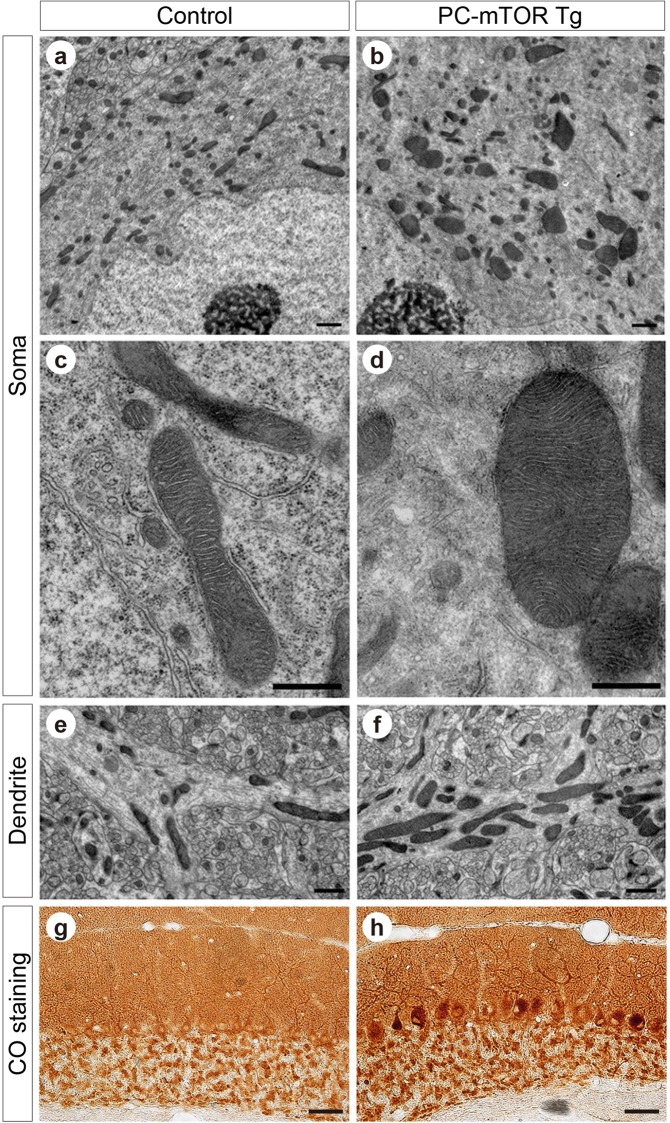
Abnormal mitochondrial morphology and activity in PC-mTOR Tg mice. (**a**–**f**) Electron microscopic images of Purkinje cells of the control and PC-mTOR Tg mice. Enlarged mitochondria were observed in both cell bodies (**a** and **b**) and dendrites (**e** and **f**). High magnitude images of cell bodies are displayed in **c** and **d**, demonstrating that the internal lamellar structure of cristae was preserved even in the enlarged mitochondria in PC-mTOR Tg mice. (**g**,**h**) Enhanced mitochondrial respiratory activity in PC-mTOR Tg mice. Enzymatic activity of cytochrome c oxidase was visualized in the cerebellar slices from the control and PC-mTOR Tg mice. Scale bars, 1 μm (**a**,**b**,**e** and **f**); 500 nm (**c** and **d**); 50 μm (**g** and **h**).

We explored the molecular mechanisms underlying Purkinje cell death by mTORC1 hyperactivation in PC-mTOR Tg mice. Activation of mTORC1 enhances the mitochondrial biogenesis by forming a complex with PGC1α and YY1^24^. We observed mitochondrial morphology in Purkinje cells by using the electron microscopy (Fig. 6a–f). As expected, the remarkably enlarged mitochondria were often found in PC-mTOR Tg mice in both cell bodies (Fig. 6a and b) and dendrites (Fig. 6e and f) compared to control mice. Despite their abnormal morphology, the internal lamellar structure of cristae was almost preserved even in enlarged mitochondria in PC-mTOR Tg mice (Fig. 6c and d). To test the mitochondrial respiratory activity, the cytochrome c oxidase activity was visualized in the cerebellar slices. Mitochondrial activity was detected in both molecular and Purkinje cell layers of control mice (Fig. 6g and h). Although similar staining patterns were also observed in PC-mTOR Tg mice, the cell bodies of Purkinje cells were stained more densely than control mice. Thus, despite their abnormal morphology, the mitochondrial respiratory function was not impaired but rather enhanced in Purkinje cells of PC-mTOR Tg mice.

**Figure 7:**
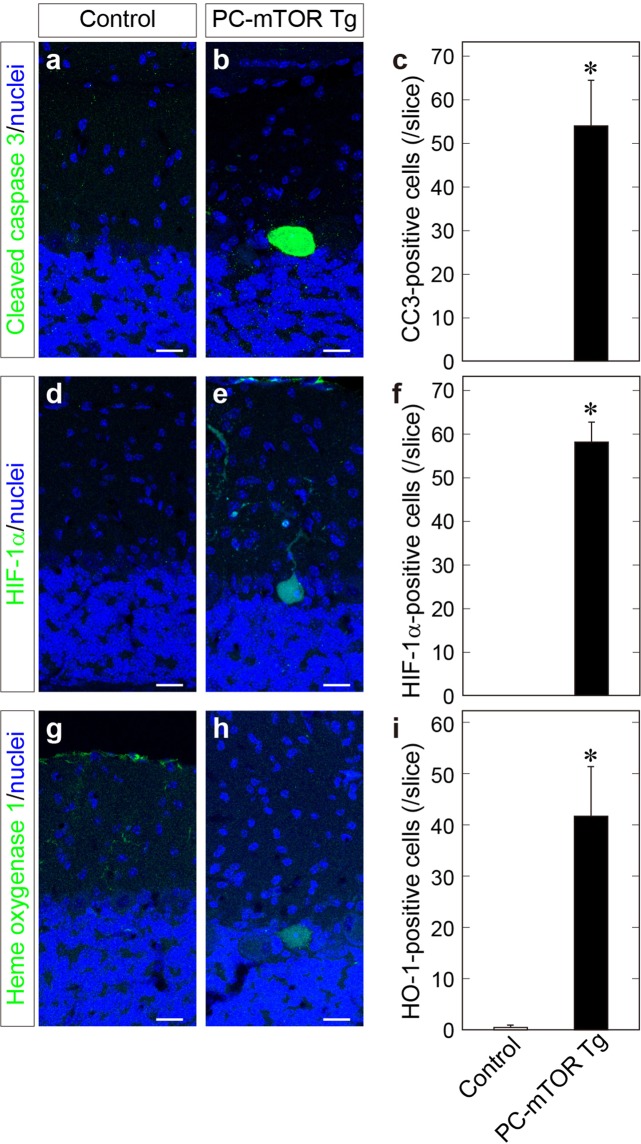
Apoptotic cell death and pseudohypoxic state of Purkinje cells in PC-mTOR Tg mice. Immunohistochemical staining of cerebellar slices using cleaved caspase 3 (**a** and **b**), HIF-1α (**d** and **e**) and heme oxygenase 1 antibodies (**g** and **h**). Elevated expression of HIF-1α and heme oxygenase 1 indicates that hyperactivation of mTORC1 induced adaptive response to hypoxia. The number of immunopositive Purkinje cells were quantified in each slice (**c**,**f** and **i**). Values are means ± SD. *p < 0.001 by Student *t*-test; control, n = 6 slices from 3 mice (**c**,**f** and **i**); PC-mTOR Tg, n = 6 slices (**c**), 10 slices (**f**) and 7 slices (**i**) from 3 mice. Scale bars, 20 μm.

In our previous study, we have shown that cortical microcephaly by hyperactivation of mTORC1 in the embryonic cortex is due to excessive apoptosis of cortical progenitors, accompanied by elevated expression of both hypoxia-inducible factor (HIF) −1α and its downstream gene^5^. We thus performed immunohistochemical analysis of cerebellar slices and found that Purkinje cells of PC-mTOR Tg mice were positive for cleaved caspase 3 (CC3), indicative of apoptotic cell death by hyperactivation of mTORC1 signaling (Fig. 7a–c). Next, we examined the expression level of HIF-1α which is upregulated by hypoxic stress and implicated in apoptotic signaling^25,26^. HIF-1α protein is also functions as a mTOR-regulated downstream effector by being stabilized by forming a complex with mTORC1^27^. We found that, in PC-mTOR Tg cerebellum, the number of HIF-1α-positive Purkinje cells was significantly increased, whereas these immunopositive cells were rarely found in the control cerebellum (Fig. 7d–f). We also confirmed the expression of HIF-1 target gene, heme oxygenase-1 (HO-1)^28^, which is often used as an oxidative stress marker. Similar to HIF-1α, upregulation of HO-1 expression was frequently found in Purkinje cells of PC-mTOR Tg mice (Fig. 7g–i). Thus, hyperactivation of mTORC1 may induce a pseudohypoxic state in Purkinje cells by activating the adaptive response to hypoxia.

We further tested whether hyperactive mTOR-induced phenotypes can be reversed by inhibiting the HIF-1 activity. PX-478 is an antitumor drug which lowers expression level of HIF-1α at both transcription and translation levels^29^. We also observed partial reduction of phosphorylation of S6 protein in Purkinje cells by PX-478 (Supplementary Fig. S2b). Administration of PX-478 to PC-mTOR Tg mice significantly ameliorated both hypertrophy and apoptosis of Purkinje cells (Supplementary Fig. S5). Together, these data indicate that mTORC1 hyperactivation in Purkinje cells elicits abnormal mitochondrial activity and cellular stresses, disrupting cellular and mitochondrial homeostasis which may result in the apoptotic cell death partially via HIF-1 signaling.

## Discussion

In this study, we demonstrate that mTORC1 signaling plays a pivotal role in cell size regulation and survival in cerebellar Purkinje cells. Several animal models for mTOR hyperactivation in Purkinje cells have been generated by deleting *Tsc1* or *Tsc2* gene, a negative regulator of mTORC1^19,20^. These Purkinje cell-specific *Tsc1/2* knockout mice exhibit behavioral abnormalities in the three chamber test, suggesting that TSC1/2-regulated signaling in Purkinje cells may be responsible for autistic-like social abnormality that was not observed in our PC-mTOR Tg mice. Since Purkinje cells undergo apoptotic cell death in both *Tsc1/2* knockout and PC-mTOR Tg mice, decreased number of Purkinje cells should not be a critical determinant of impaired social behaviors. Moreover, Purkinje cell apoptosis was observed much earlier in PC-mTOR Tg mice (3 weeks of age, Figs 1h,i and 7a–c) compared to *Tsc1/2* knockout mice (2 months of age), suggesting that mTORC1 activity may be higher in PC-mTOR Tg than *Tsc1/2* knockout mice. Therefore, higher activity of mTORC1 alone is considered to be insufficient for the onset of autistic-like symptoms. This notion is supported by previous reports in mouse and human. Purkinje cell-specific Raptor knockout mice show abnormal social interaction^18^, suggesting that loss of mTORC1 activity in Purkinje cells induces autistic behavior. In addition, in some clinical cases of human tuberous sclerosis, changes in GAP activity of Rheb by *TSC1/2* mutations is not always correlated with disease severity of patients with tuberous sclerosis^13^, implicated in hyperactive mTORC1-independent symptoms.

The difference in mTORC1 activity between *Tsc1/2* knockout and PC-mTOR Tg mice can be partly attributable to the different regulatory mechanisms of mTORC1 signaling. The mTORC1 signaling is regulated by many signaling molecules such as AMP kinase and Rag GTPases, independent of TSC1/2 complex^30–32^. These signaling modulators should influence mTORC1 activity in *Tsc1/2* knockout mice. On the other hand, the level of mTORC1 activation is determined directly by the amount of hyperactive mTOR expression from transgene in PC-mTOR Tg mice. Therefore, in PC-mTOR Tg mice, specific and potent activation of mTORC1 by hyperactive mTOR should provide a disease model that focuses on the contribution of mTORC1 in Purkinje cells.

We found that sustained activation of mTORC1 signal impaired Purkinje cell maturation and disrupted the establishment of CF mono-innervation (Fig. 5d). As for mTOR-related molecule, multiple CF innervation is observed in Purkinje cell-specific knockout of Rictor, a component which is essential for mTORC2 activity^18^. In addition, Purkinje cells of Rictor knockout mice display increased number of the primary dendrites and self-crossing, both of which are also obvious in PC-mTOR Tg mice. Considering that mTORC2 is involved in cytoskeletal dynamics^33^, mTORC2 signaling may regulate synapse formation and dendrite arborization in developing Purkinje cells. Furthermore, the strong consistencies of these phenotypes between Rictor knockout and PC-mTOR Tg mice imply the negative feedback inhibition of mTORC2 by hyperactivated mTORC1 signaling^34^.

In parallel with the regulation of the nutrient signal, mTOR signaling is involved in homeostatic process such as cell size, osmolality and mitochondrial mass^1,2^. We observed several perturbations of maintenance of the cellular and mitochondrial homeostasis in PC-mTOR Tg mice. Increased cell size and thickened dendrites of Purkinje cells may reflect excessive protein synthesis and suppression of autophagy by hyperactivated mTORC1 signaling. The low input resistance observed in PC-mTOR Tg mice (Table 1) was partially attributable to these increased cellular mass. The excessive protein synthesis also upregulated an expression of HIF-1α and its downstream target, HO-1^28^ in PC-mTOR Tg mice (Fig. 7). Under normoxic conditions, HIF-1α is continuously degraded by the ubiquitin-proteasome system, and HIF-1α protein is also stabilized by forming a complex with activated mTORC1^27^. Therefore, hyperactivation of mTORC1 signaling induced the adaptive response to hypoxic stress, and may represent a pseudohypoxic state in PC-mTOR Tg mice, thereby resulting in apoptosis of Purkinje cells. Association of pseudohypoxia with neuronal cell death is also observed in the cortical progenitor cells expressing hyperactivated mTORC1^5^.

Dysregulated homeostasis was also found in abnormal mitochondrial mass and activity in PC-mTOR Tg mice (Fig. 6). The mTORC1 regulates mitochondrial biogenesis and oxidative function through PGC-1α. In PC-mTOR Tg mice, enlarged mitochondrial mass and enhanced respiratory function may be attributable to increased protein synthesis of mitochondrial complexes by hyperactivated mTORC1. Expanded size of mitochondria may mislocalize their synaptic distribution^35^, thereby inhibiting the synapse formation and the maturation of CF innervation to Purkinje cells in PC-mTOR Tg mice (Fig. 5d). Association of neurodegenerative diseases with the alteration of mitochondrial dynamics also supports the notion that apoptosis of Purkinje cells may be attributable to the abnormal shape and activity of mitochondria. Together, mTORC1 signal should be important for the maintenance of the cellular and mitochondrial homeostasis in Purkinje cells that may provide a molecular basis of neurological diseases.

## Methods

### Mice

Animal experiments were approved by the Institutional Animal Care and Use Committee of the University of Tokyo (Permit No. P14-117) and Hiroshima University Animal Research Committees (Permit No. A18-47), and conducted in accordance with the guidelines of The University of Tokyo and Hiroshima University. All mice were housed under a 12 h light/12 h dark cycle (light on at 8 a.m.) in cage with food and water available *ad libitum*. TRE-mTOR Tg and L7-tTA Tg mice were generated as described previously^5,16^. Genetic background of the Tg mice used in this study was a hybrid of C57BL/6 and ICR. For drug administration, doxycycline (Dox; D9891, Sigma-Aldrich) was dissolved in drinking water (200 mg/l), and supplied orally by water bottle. Administration of Dox started at the time of parent mating, and continued until sacrifice. A stock solution of rapamycin (R-5000, LC Laboratories) was prepared at 25 mg/ml in ethanol (1473-53, Nacalai Tesque), and was diluted to 1 mg/ml in 5% Tween-80 and 5% polyethylene glycol 400 as working solution. Rapamycin solution was provided intraperitoneally 3 times per week at 10 mg/kg from 3 to 6 weeks of age. A stock solution of PX-478 (202350, MedKoo Biosciences) was prepared at 40 mg/ml in dimethyl sulfoxide (D2650, Sigma) and was diluted in PBS upon administration. The dosage and timing of administration of PX-478 were the same protocol as rapamycin.

### Immunoblot analysis

Immunoblot analysis was essentially performed as described previously^5^. Cerebella were isolated from the control and PC-mTOR Tg mice from 1 to 6 weeks of age, and homogenized in the lysis buffer containing 50 mM Tris-HCl (pH 7.5), 150 mM NaCl, 1% Triton X-100, protease inhibitor (Complete EDTA-free, Roche) and phosphatase inhibitor (PhosSTOP, Roche). Protein extracts were quantitated via the Bradford assay (Coomassie Plus Protein Assay Reagent, Thermo Scientific), and subjected to Western blot analysis. Antibodies used in this study were as follows: anti-FLAG (A8592, Sigma), β-actin (A2228, Sigma). Bound antibodies were visualized by using a HRP conjugated-secondary antibody (Jackson Immunoresearch) and an enhanced chemiluminescence reagent (ECL prime Western Blotting Detection Reagent, GE Healthcare).

### Histology

Mice were deeply anesthetized and perfused with 4% paraformaldehyde in 0.1 M phosphate buffer (pH 7.4, PB). The fixed brains were cryoprotected and sectioned into 40-μm-thickness slices using the freezing microtome. Sections of 20-μm-thickness were prepared by using a cryostat (CM1850, Leica Microsystems) and mounted on MAS-coated glass slides (Matsunami Glass). Sections were immunolabeled with primary antibodies against calbindin (AB1778, Chemicon or #300, Swant), phospho-S6 (#2211, Cell Signaling Technology), cleaved caspase-3 (#9661, Cell Signaling Technology), HIF-1α (ab1, Abcam), heme oxygenase-1 (ab13248, Abcam) and VGluT2 (kind gifted from Prof. Masahiko Watanabe at Hokkaido University). Bound antibodies were visualized with Alexa-488 or Alexa-633 conjugated-secondary antibody (Life Technologies) and TO-PRO-3 were used for nuclear staining (Life Technologies). The sections were coverslipped with Vectashield Mounting Medium (H-1000, Vector Laboratories), and viewed by a confocal laser scanning microscope (TSC-SP5II, Leica Microsystems). For cytochrome oxidase histochemistry, sections were washed with PBS, and incubated for 4 h at 37 °C in a solution containing cytochrome c (3 mg, Sigma), 3,3-diaminobenzidine (5 mg, Sigma), and sucrose (450 mg) in 10 ml of 0.1 M PB.

### Behavioral analysis

All behavioral analyses start at noon. Prior to each experiment, test mice were habituated by handling the mice for 5 min per day for one week.

Footprint test was performed to assess the walking pattern and gait of the mice. The hind paws of mice were coated with non-toxic ink, and footprints were traced on the paper covering a runway of about 10 cm width and 42 cm length. Footprint trials were performed three times for each mouse. The footprint patterns were scanned, and the stride widths of hind limbs were measured using ImageJ.

An accelerating rotarod (MK-660C, Muromachi Kikai) was used to measure motor coordination and learning. Mice were tested for 5 consecutive days, and 3 trials per day with 15 min inter-trial interval. Test mice were placed on a rotating rod, with a 30 mm diameter, that accelerated from 4 to 40 rpm at 300 s. The maximum observation time was 300 s. The latency to fall from the rod was recorded and the mean value was calculated for each day for each mouse. Weight of each mouse was recorded every day after rotarod test.

Open field test was used to measure anxiety-like behavior and locomotor activity. The open field consisted of a 400 mm height, 500 mm diameter with polyvinyl chloride (OF-25M, Muromachi Kikai). Each mouse was placed on in the center of the open field and allowed to explore freely for 10 min. During the 10 min test session, locomotion of each mouse was monitored using CCD camera (XC-117, SONY) mounted above the field. Recorded movies were analyzed by tracking software (CompACT VAS, Muromachi Kikai) to calculate total time traveled, total distance traveled and the time spent in the periphery or center of the field.

The three chamber apparatus (BS-402260, BrainScience Idea) was a transparent Plexiglas box (620 mm width × 410 mm height × 225 mm depth) separated into three chambers (each 200 mm width × 400 mm height ×220 mm depth) by transparent plates with doors (50 mm width × 80 mm height). A cylindrical wire cage (100 mm diameter × 175 mm height; BS-402260-02, BrainScience Idea) was used as an object or the cage housing a stranger mouse, and placed in left and right side chambers. The three chamber apparatus and wire cage were cleaned with 70% ethanol and wiped with KimWipes between each test. The three chamber test consisted of two phases, habituation and sociability phases. During the habituation phase, the test mouse was placed in the center chamber and was allowed to explore freely to all three chambers and two empty cylindrical wire cages for 10 min. During the sociability phase, a stranger male ICR (CD-1) mouse that had never been contacted with the test mouse, was placed in one of the empty wire cage, and the wire cage on the other side remained empty as an inanimate object. The test mouse was placed in the center chamber and was allowed to explore all of the three chambers for 10 min. The activity of the test mouse was recorded using digital video camera (HHR-PJ760, Sony). The time spent in each chamber was manually measured.

### Electrophysiology

Parasagittal cerebellar slices (250 μm in thickness) were prepared from 7–8 weeks-old mice as described previously^7,36^. In brief, mice were deeply anaesthetized by CO_2_ inhalation and decapitated. The brain was quickly removed and parasagittal cerebellar slices were cut with a vibratome slicer (VT1000S; Leica) in chilled normal ACSF containing (in mM) 125 NaCl, 2.5 KCl, 2 CaCl_2_, 1 MgSO_4_, 1.25 NaH_2_PO_4_, 26 NaHCO_3_, and 20 glucose, bubbled with 95% O_2_ and 5% CO_2_. After cutting, the slices were recovered for 30 min at 35 °C, and then kept for up to 6 h at 25 °C in the normal ACSF.

Whole-cell recordings were made from visually identified Purkinje cells using an upright microscope (BX50WI, Olympus). All data were recorded at 32 °C with an EPC10 patch clamp amplifier (HEKA). Online data acquisition and offline data analysis were performed using Patch Master software (HEKA) and Fit Master software (HEKA). Pipette solution for current clamp recordings composed of (in mM) 125 K-methanesulfonate, 10 KCl, 5 NaCl, 10 HEPES, 0.5 EGTA, 4 MgCl_2_, 4 ATP, 0.4 GTP, and 15 biocytin (pH 7.3, adjusted with KOH). Resting membrane potentials were measured immediately after the establishment of whole-cell configuration. To evaluate electrical membrane properties of Purkinje cells, hyperpolarizing and depolarizing currents (from −500 pA to 1000 pA with increments of 100 pA) of 700 ms duration were applied from a membrane potential of −65 mV. The firing rate was calculated from spike numbers elicited by each depolarizing current injection. Input resistance was calculated by averaging resistances calculated from voltage deflections resulting from −300, −400 and −500 pA current injections. Pipette solution for voltage clamp recordings composed of (in mM) 50 CsCl, 10 CsD-gluconate, 20 TEA-Cl, 20 BAPTA, 4 MgCl_2_, 4 ATP, 0.4 GTP, 30 HEPES, and 15 biocytin (pH 7.3, adjusted with CsOH). Picrotoxin (100 μM) was always supplemented to the normal ACSF to block inhibitory synaptic transmission during recordings. Stimulation pipettes were filled with the normal ACSF and used to apply square pulses for focal stimulation (duration, 0.1 ms; amplitude, from 0 V to 90 V). For CF stimulation, pulses were given at 0.2 Hz, and the stimulation pipette was systematically moved around the Purkinje cell soma in the granule cell layer. The number of CFs innervating the recorded Purkinje cell was estimated as the number of discrete CF-EPSC steps during gradually increasing the stimulus intensity. Data in the text and Figures were expressed as mean ± SEM. Statistical evaluations were performed using Sigmaplot 12.5 software (Systat software).

### Electron microscopy

Under deep pentobarbital anesthesia (100 mg/kg of body weight), three mice at 4 weeks of age for each experimental group were perfused transcardially with 2% paraformaldehyde/2% glutaraldehyde in 0.1 M PB. Cerebellar slices were further post-fixed with 1% osmium tetroxide in 0.1 M PB for 2 h, stained with 2% uranyl acetate for 1 h, dehydrated in graded alcohols, and embedded in Epon 812 resin. Ultrathin sections (75 nm in thickness) were cut with a Leica Ultracut. Electron microscopic images were taken with an H7650 electron microscope (Hitachi).

## Supplementary information

Supplementary Information
