# Supplementary material for: Hyperactivation of mTORC1 disrupts cellular homeostasis in cerebellar Purkinje cells
Source: Sci Rep. 2019 Feb 26;9:2799. doi: 10.1038/s41598-019-38730-4 (PMC6391425; doi:10.1038/s41598-019-38730-4)

Supplementary Information

Hyperactivation of mTORC1 disrupts cellular homeostasis in cerebellar Purkinje cells.

Yusuke Sakai[1], Hidetoshi Kassai[1]*, Hisako Nakayama[2,3,†], Masahiro Fukaya[4], Tatsuya Maeda[5,‡], Kazuki Nakao[1], Kouichi Hashimoto[3], Hiroyuki Sakagami[4], Masanobu Kano[2,6], Atsu Aiba[1]*

[1]*Laboratory of Animal Resources, Center for Disease Biology and Integrative Medicine, Graduate School of Medicine, The University of Tokyo, Tokyo 113-0033, Japan.*

[2]*Department of Neurophysiology, Graduate School of Medicine, The University of Tokyo, Tokyo 113-0033, Japan.*

[3]*Department of Neurophysiology, Graduate School of Biomedical and Health Sciences, Hiroshima University, Hiroshima 734-8551, Japan.*

[4]*Department of Anatomy, Kitasato University School of Medicine, Sagamihara 252-0374, Japan.*

[5]*Institute of Molecular and Cellular Biosciences, The University of Tokyo, Tokyo 113-0032, Japan.*

[6]*International Research Center for Neurointelligece (WPI-IRCN), The University of Tokyo Institutes for Advanced Study (UTIAS), The University of Tokyo, Tokyo 113-0033, Japan.*

[†]*Present address: Department of Physiology I (Neurophysiology), School of Medicine, Tokyo Women's Medical University, Tokyo 162-8666, Japan.*

[‡]*Present address: Department of Biology, Hamamatsu University School of Medicine, Hamamatsu, Shizuoka 431-3192, Japan.*

*Correspondence and requests for materials should be addressed to A.A. (e-mail: aiba@m.u-tokyo.ac.jp) or H.K. (e-mail: kassai@m.u-tokyo.ac.jp)

Legend for Supplementary Figures

Supplementary Figure S1 Generation of hyperactive mTOR Tg mice. **a,** Schematic drawing of transgenic strategy for Purkinje cell-specific expression of hyperactive mTOR. DNA fragments encoding hyperactive mTOR is placed downstream of TRE promoter, and transactivator tTA is expressed under the control of Purkinje cell-specific L7 promoter. **b,** Expression of active mTOR in the cerebellum. Cerebellar lysates were prepared from control and PC-mTOR Tg mice at designated ages, and immunoblotted with antibody to FLAG tag. β-actin antibody was used as a loading control. Active mTOR expression was clearly observed at 2-4 weeks of age. However, this expression was decreased at 5 weeks of age and almost diminished at 6 weeks of age, probably due to decreased number of Purkinje cells by apoptotic cell death. **c,** Full-length blot used in (b).

Supplementary Figure S2 Phosphorylation of S6 protein after drug treatments. Mice were intraperitoneally received rapamycin (a) or PX-478 (b) from 3 to 6 weeks of age, and subjected to the immunohistochemical analysis using an antibody against pS6. Scale bars, 20 μm. Values are means ± SD. $*p < 0.001$ by one-way ANOVA with Tukey; n = 15 cells from 2 mice (control, PC-mTOR Tg and PC-mTOR Tg +rapa) (a). $*p < 0.001$, $**p < 0.05$ by one-way ANOVA with Tukey; n = 15 cells from 2 mice (control and PC-mTOR Tg +PX-478); n = 11 cells from 2 mice (PC-mTOR Tg) (b). n.s., not significant.

Supplementary Figure S3 Behavioral analyses of PC-mTOR Tg mice. **a,** Open field test. Compared to the control, PC-mTOR Tg mice showed significant hypoactivity in time (left panel) and distance (center panel) traveled in

the open field. No significant difference was observed in time spent in either center or peripheral region of the open field (right panel). **b,** Three chamber test. In the habituation phase, there was no difference in the preference between left and right chambers. In the sociability phase, control mice spent more time in the left chamber housing a stranger mouse than the right chamber with an empty cage. This trend was also observed in PC-mTOR Tg mice, indicating that the social behavior is not affected by hyperactivation of mTORC1 signaling in Purkinje cells. Values are mean ± SD. $*p < 0.01$ and $**p < 0.05$ by Student *t*-test; control, n = 8; PC-mTOR Tg, n = 12. n.s., not significant.

Supplementary Figure S4 Immunohistochemical analysis of climbing fiber innervations to Purkinje cells. **a, b,** Immunohistochemical images of the cerebellum showing distribution of VGluT2 (green) and calbindin (red). CF terminals can be labeled with VGluT2 immunoreactivity. Scale bars, 50 μm. **c, d,** Quantification of molecular layer thickness and CF innervation in Purkinje cells. Molecular layer thickness of PC-mTOR Tg mice was significantly decreased compared to the control (c), probably due to progressive loss of Purkinje cells. The height of VGluT2-positive CF terminals relative to the thickness of molecular layer of Purkinje cells was decreased in PC-mTOR Tg mice (d), indicative of abnormal CF innervation to Purkinje cells. Values are mean ± SD. $*p < 0.01$ by Student *t*-test; control, n = 30 cells from 2 mice; PC-mTOR Tg, n = 27 cells from 2 mice.

Supplementary Figure S5 Partial rescue of hypertrophy and apoptosis of PC-mTOR Tg mice by inhibition of HIF-1 signaling. **a,** Confocal microscopic images of cerebellar slices stained with calbindin antibody (green).

Administration of PX-478 from 3 to 6 weeks of age partially reversed the phenotype induced by hyperactivation of mTORC1. **b, c,** Quantification of density and soma size of Purkinje cells from the control and PC-mTOR Tg mice. Values are mean ± SD. $*p < 0.01$ by one-way ANOVA with Tukey; control, n = 8 slices from 2 mice; PC-mTOR Tg, n = 8 slices from 2 mice; PC-mTOR Tg + PX-478, n = 13 slices from 3 mice (b); control, n = 543 cells from 2 mice; PC-mTOR Tg, n = 342 cells from 2 mice; PC-mTOR Tg + PX-478, n = 276 cells from 3 mice (c).

Supplementary Figure S1

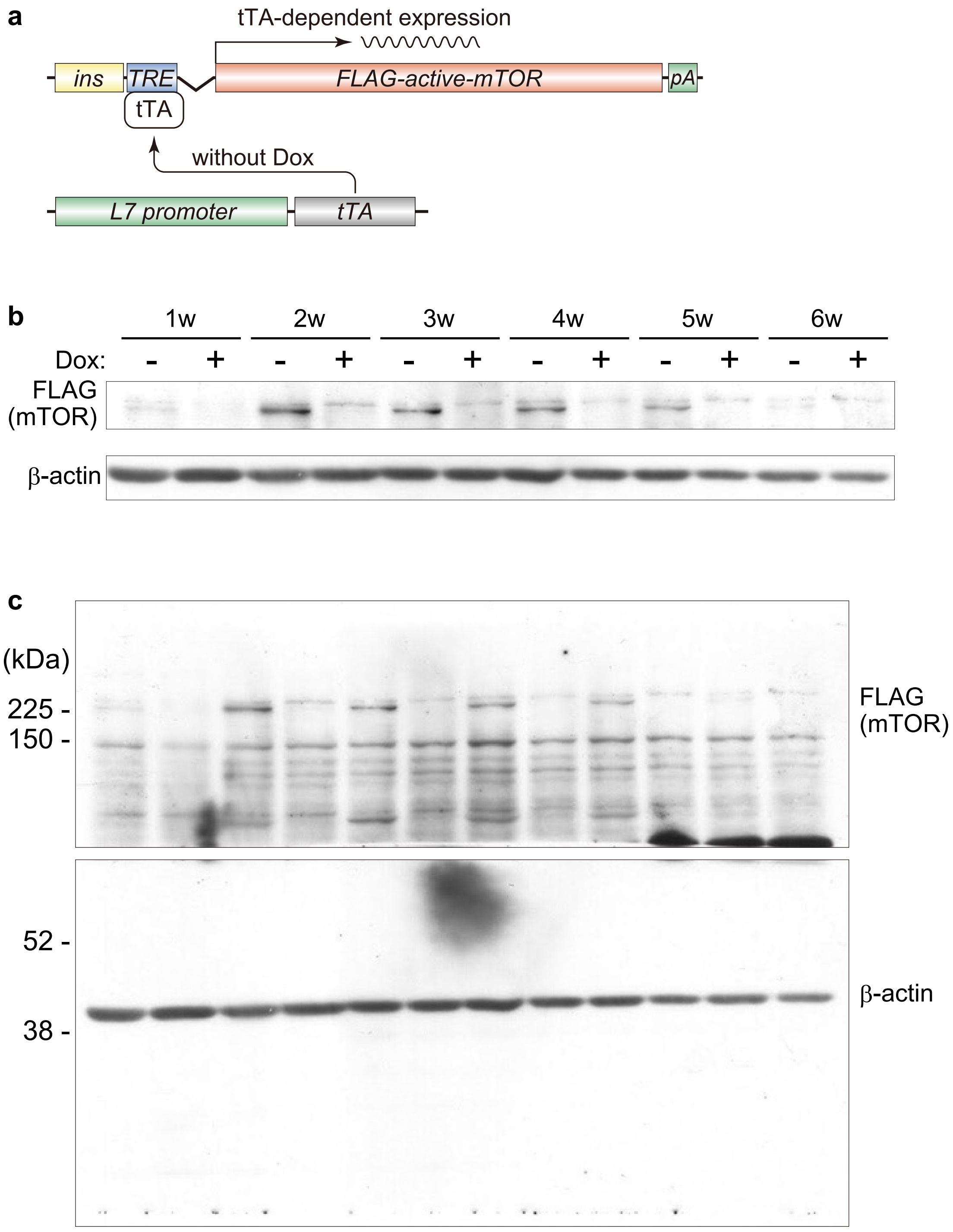

Supplementary Figure S2

a

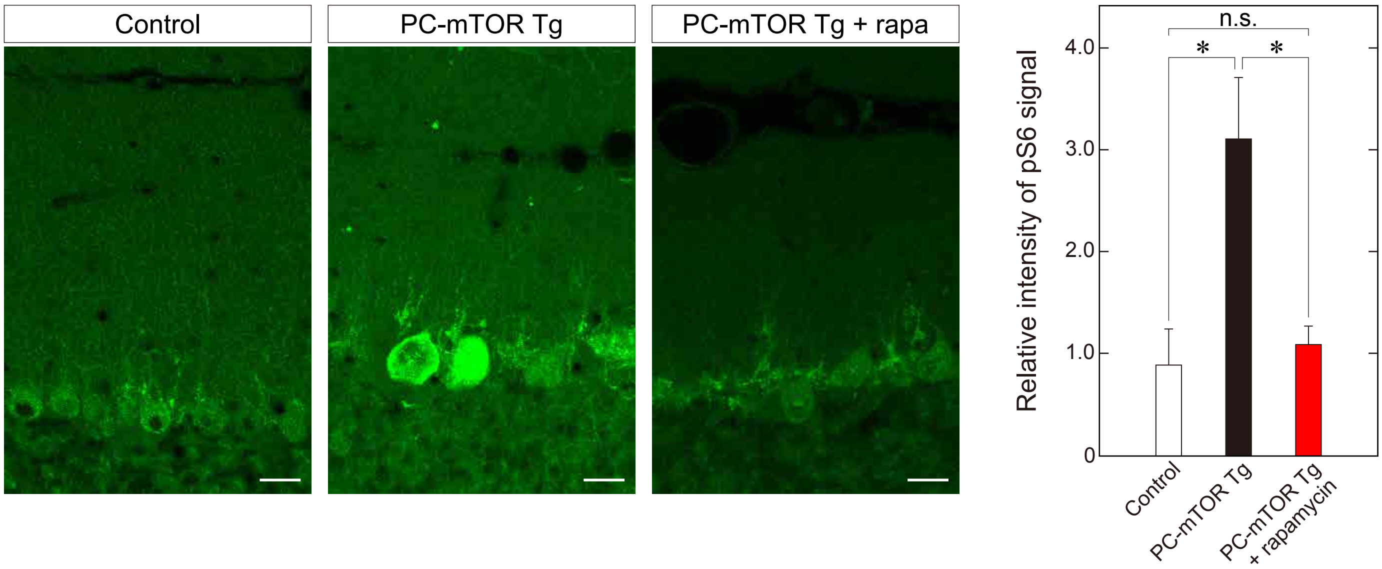

b

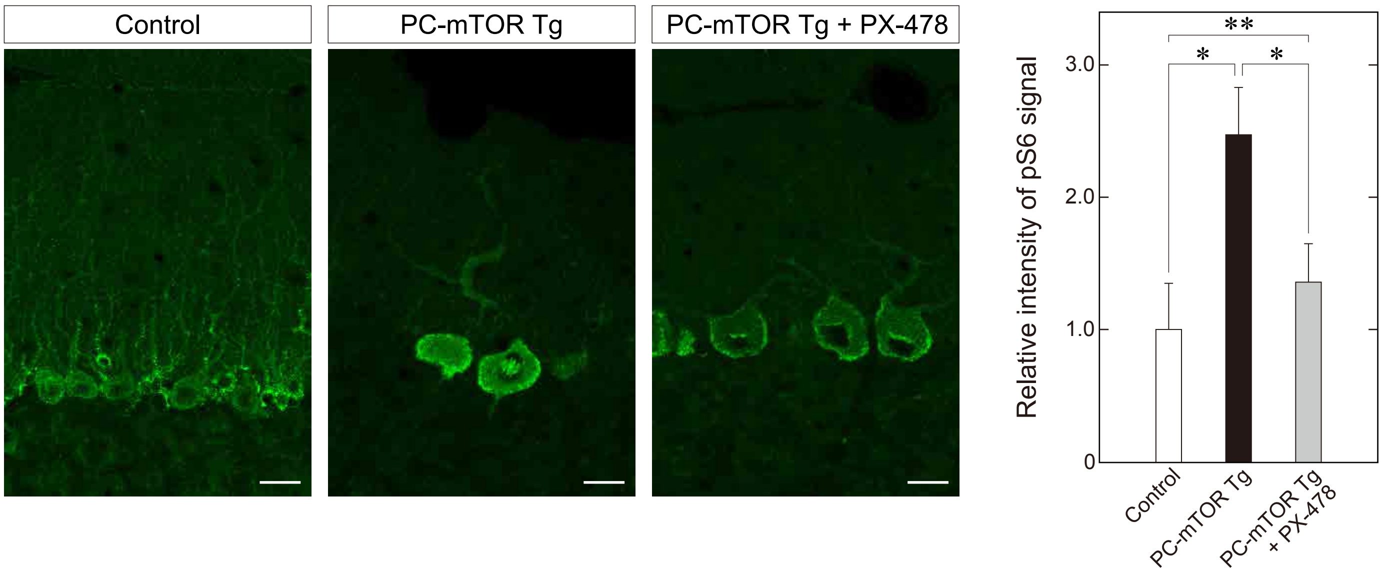

Supplementary Figure S3

a, Open field test

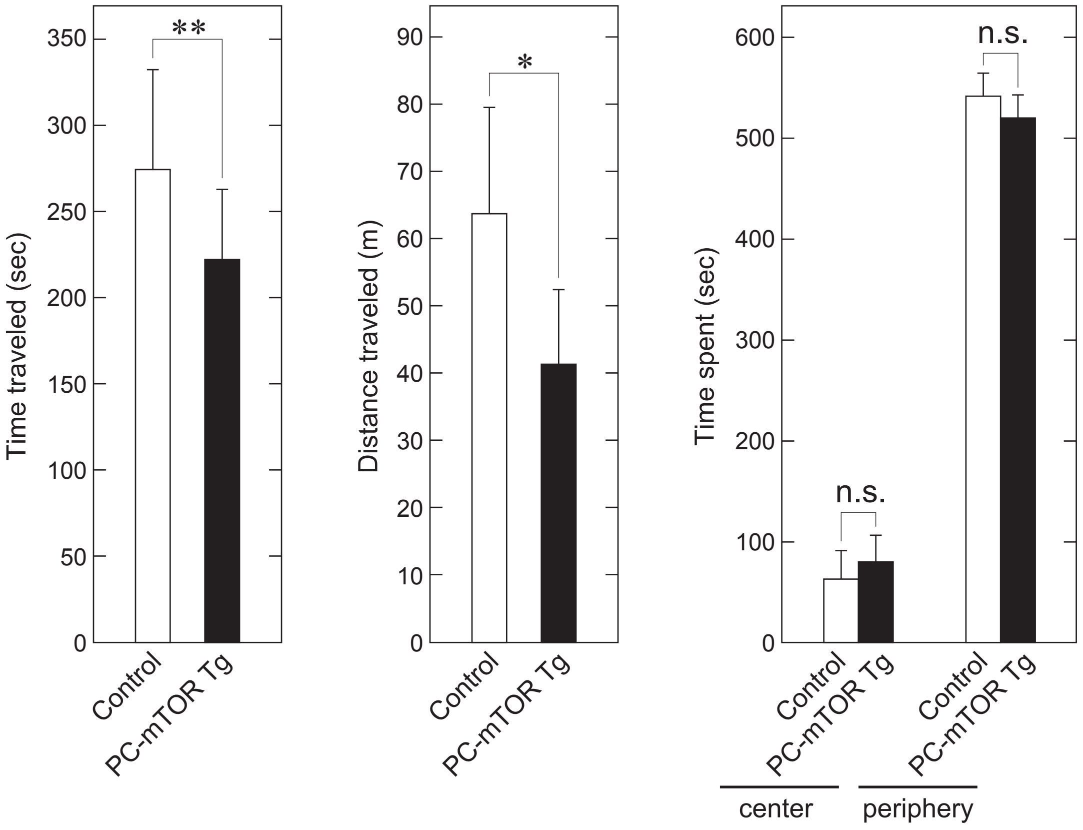

b, Three chamber test

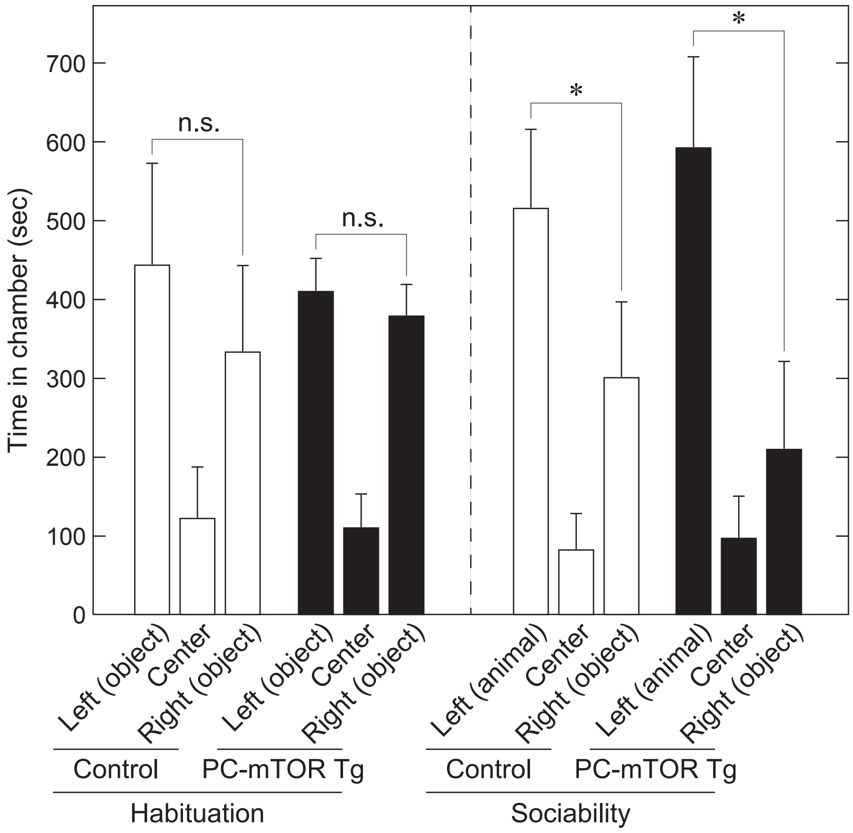

Supplementary Figure S4

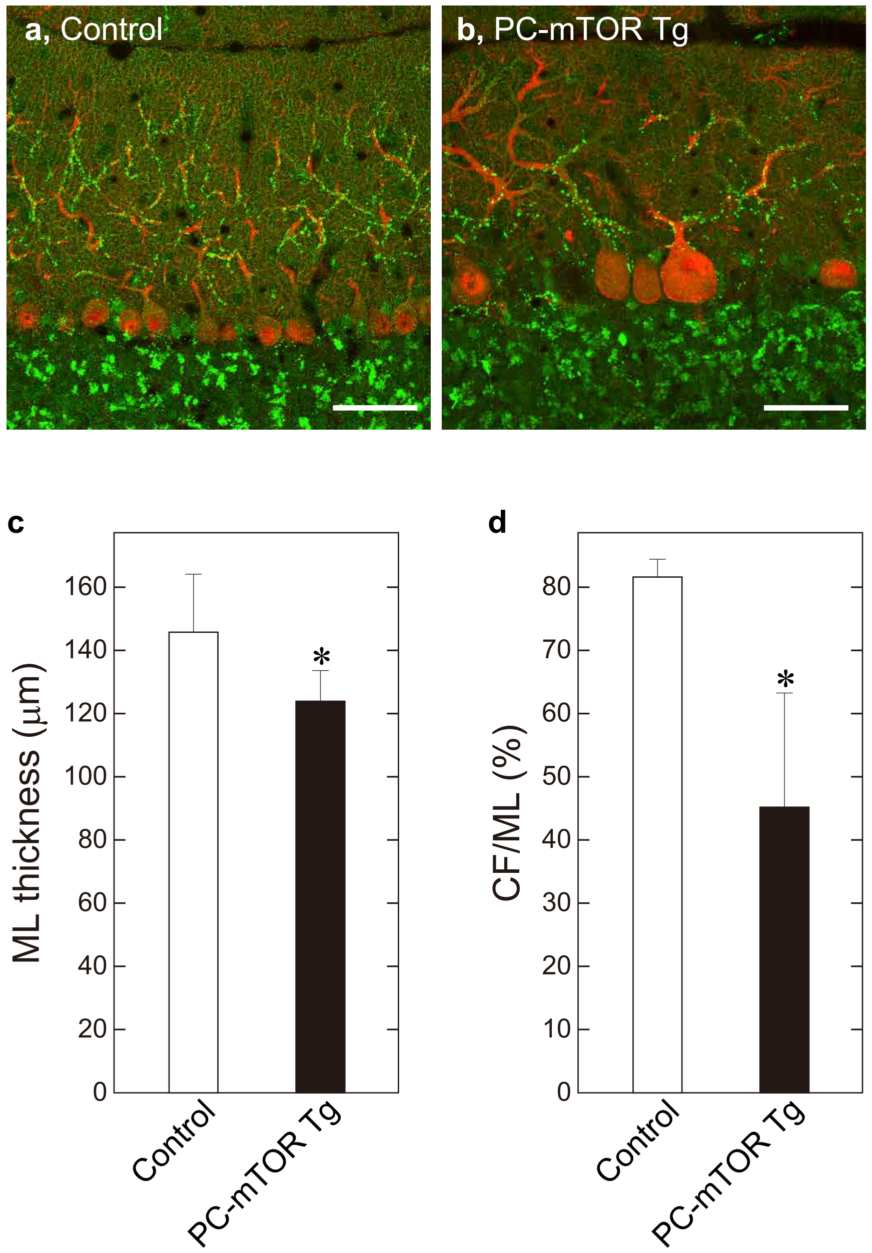

Supplementary Figure S5

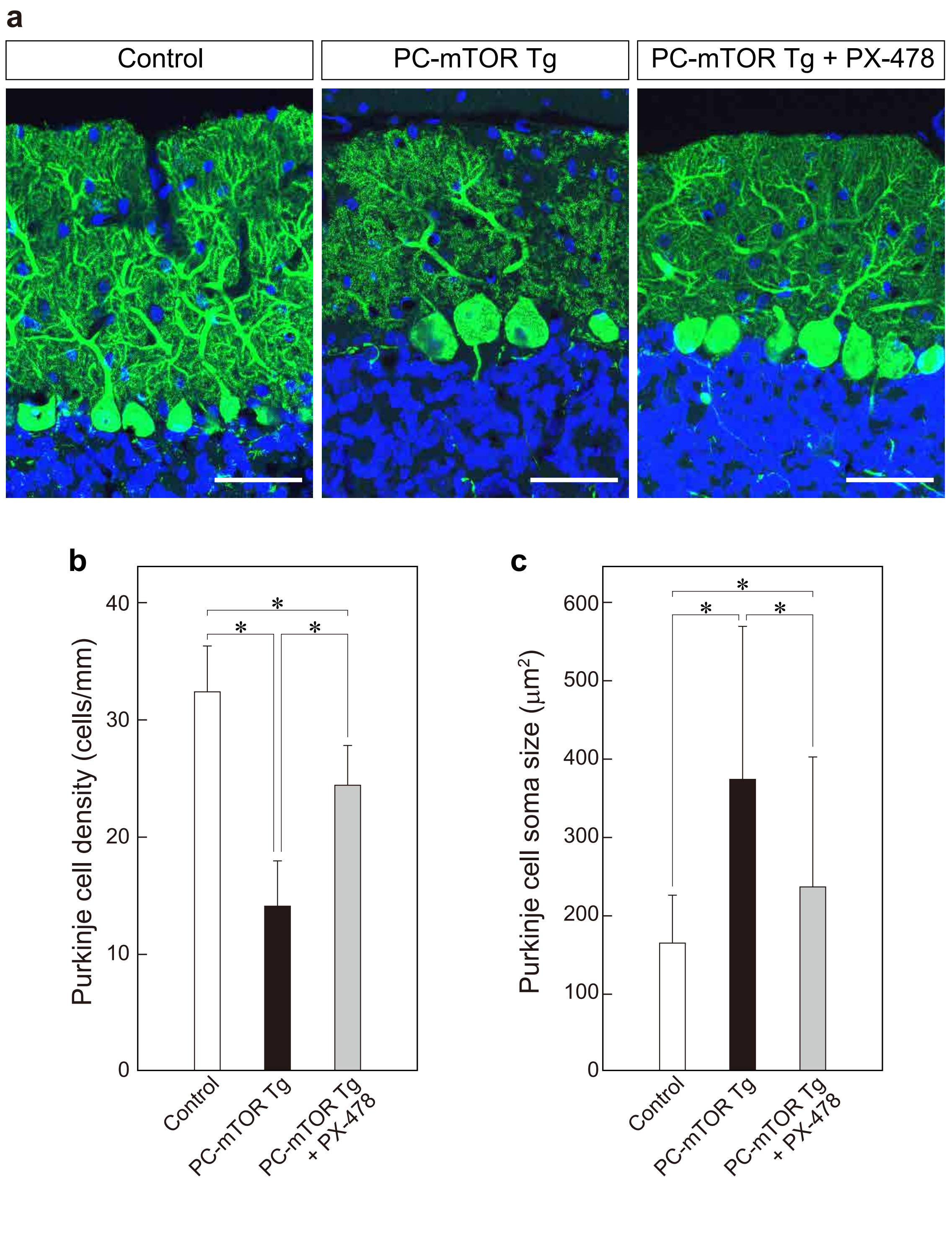

Supplement: Supplementary file 1 — Supplementary Information [file 41598_2019_38730_MOESM1_ESM.pdf]
